# EBV-associated post-transplantation B-cell lymphoproliferative disorder following allogenic stem cell transplantation for acute lymphoblastic leukaemia: tumor regression after reduction of immunosuppression - a case report

**DOI:** 10.1186/1746-1596-5-21

**Published:** 2010-03-31

**Authors:** Alexander Krenauer, Alexander Moll, Wolfram Pönisch, Nicole Schmitz, Gerald Niedobitek, Dietger Niederwieser, Thomas Aigner

**Affiliations:** 1 Institute of Pathology, University of Leipzig, Liebigstr. 26, 04103 Leipzig, Germany; 2 Division of Hematology and Oncology, University of Leipzig, Johannisallee 32, 04103 Leipzig, Germany; 3 Institute of Pathology, Sana Klinikum Lichtenberg and Unfallkrankenhaus Berlin, Fanningerstr. 32, 10365 Berlin, Germany

## Abstract

Epstein-Barr virus (EBV)-associated B-cell post-transplantation lymphoproliferative disorder (PTLD) is a severe complication following stem cell transplantation. This is believed to occur as a result of iatrogenic immunosuppression leading to a relaxation of T-cell control of EBV infection and thus allowing viral reactivation and proliferation of EBV-infected B-lymphocytes. In support of this notion, reduction of immunosuppressive therapy may lead to regression of PTLD.

We present a case of an 18-year-old male developing a monomorphic B-cell PTLD 2 months after receiving an allogenic stem cell transplant for acute lymphoblastic leukemia. Reduction of immunosuppressive therapy led to regression of lymphadenopathy. Nevertheless, the patient died 3 months afterwards due to extensive graft-vs.-host-disease and sepsis. As a diagnostic lymph node biopsy was performed only after reduction of immunosuppressive therapy, we are able to study the histopathological changes characterizing PTLD regression. We observed extensive apoptosis of blast cells, accompanied by an abundant infiltrate comprising predominantly CD8-positive, Granzyme B-positive T-cells. This observation supports the idea that regression of PTLD is mediated by cytotoxic T-cells and is in keeping with the observation that T-cell depletion, represents a major risk factor for the development of PTLD.

## Introduction

The Epstein-Barr virus (EBV), a human herpes virus, was first discovered in 1964 in cultured tumour cells from Burkitt lymphoma [1]. Subsequently, EBV was shown to be ubiquitously distributed, infecting over 90% of the adult human population world wilde. Upon primary infection, B-cells are immortalized and driven into proliferation. Viral infection in B-cells usually remains latent, i.e., infectious virus is not produced, and is characterised by the expression of several viral proteins, notably EBV-encoded nuclear antigen (EBNA) 2 and latent membrane protein (LMP) 1, which are thought to orchestrate virus-induced immortalisation and proliferation of B-cells. As a consequence, EBV-specific cytotoxic (CD8-positive) T-cells are generated which control EBV infection and lead to the establishment of an asymptomatic life-long virus persistence in memory B-cells with minimal viral gene expression. This, however, can change in transplant recipients in whom iatrogenic immunosuppression leads to a relaxation of T-cell control of EBV infection allowing the re-emergence of proliferating EBV-infected B-cells and leading to post-transplantation lymphoproliferative disorders (PTLDs) (for review see Hsieh 1999 and Loren 2003 [2,3]). According to the WHO classification PTLD comprise five subtypes, i.e., early lesions (plasmacytic hyperplasia and infectious mononucleosis-like PTLD), polymorphic PTLD, monomorphic B-cell PTLD, monomorphic T/NK-cell PTLD and classical Hodgkin lymphoma-like PTLD with the first two representing EBV-driven B-cell proliferations [4] of poly- and monoclonal origin. Risk factors for the development of a PTLD after HSCT (human stem cell transplantation) include T-cell depletion, age, HLA-mismatch, specific antilymphocyte anti-graft-versus-host disease therapies, splenectomy and HSCT for primary immunodeficiency disorders [2,3]. The treatment options for B-cell PTLDs include reduction of immunosuppression, antiviral therapy, interferon alpha therapy, CD20 antibody therapy and chemotherapy.

In this report, we present a case of EBV-associated PTLD following allogenic stem cell transplantation for acute lymphoblastic leukemia with evidence of tumor regression subsequent to reduction of immunosuppression, showing for the first time the histopathological changes within lymph nodes after reduction of immunosuppression.

## Clinical Case

An 18-year-old male patient presented with tiredness, night sweats, dyspnoea at exercise and shivering. The blood count showed anemia (Hb 3,1 mmol/l and thrombocythopenia (thrombocytes 143/nl). The white blood cell count and the differential blood smear were normal (leukocytes 6800/μl: granulocytes 60%; lymphocytes 36%; monocytes 2%; eosinophils 2%; blasts not detectable).

A diagnosis of acute lymphoblastic leukemia (L2 according to the FAB classification) was made based on bone marrow trephine biopsy showing dense lymphoblastic infiltrates (about 90%) with a severely reduced residual haematopoiesis. The tumor cells were positive for CD19 and cytoplasmatic IgM as well as CD10, CD34 and TdT consistent with ALL. The myeloid marker CD13 was aberrantly coexpressed. No expression was observed for CD3. FACS analysis showed predominantly immature B-lymphocytes, strongly positive for CD34 and CD10 confirming the diagnosis of a common-B-ALL. PCR-analysis revealed no evidence of BCR-ABL fusion transcripts.

Immediately after diagnosis, primary chemotherapy was started with dexamethasone, cyclophosphamide and methotrexate 15 mg intrathecally for 5 days. Subsequently, an induction chemotherapy phase I according to the GMALL 07/2003 protocol (German Multicenter Study Group for Adult ALL) was performed with dexamethasone, vincristine, daunorubicine and asparaginase. During the induction therapy a prophylactic irradiation of the central nervous system was performed (24 Gy). Complete remission was achieved. However, since FACS analysis of the bone marrow showed a residual common-B-ALL population of 2%, consolidation therapy (according to GMALL) was completed and an unrelated allogenic stem cell donor was identified (HLA-status: A, B, DRB1, DQB1 identical, C: mismatch).

Eight months after the initial diagnosis, allogenic peripheral stem cell transplantation was performed: the conditioning regimen consisted of 12Gy total body irritation in 6 fractions with shielding of the lungs (10Gy), cyclophosphamide dose 60 mg/kg/d at 2 days and ATG (rabbit) 1000 mg/d = 14,7 mg/kg/d at day -4 to -1 before first PSCT. Graft-versus-host prophylaxis consisted of cyclosporin A, MTX and prednisolon. Whereas no early complications were noted, mucositis later required parenteral alimentation. Because of fever of unknown origin the patient was treated with antibiotics for 28 days after transplantation, when the patient was referred to a rehabilitation center.

Seven weeks post-transplantation, the patient developed fever (up to 39°C) and a rapidly progressing painful lymphadenopathy at multiple sites (submandibular, axillar and inguinal). CrP and creatinine increased and he developed leuko- and thrombocytopenia: He was therefore was referred to the hospital for further diagnosis. Medication at transferral included diflucane^® ^200 (fluconazol 200 mg) twice a day i.v. and ceftriaxone 2 g i.v. per day. The dose of sandimmune^® ^optoral was reduced from 150 mg to 100 mg upon arrival at the hospital. An infection with EBV and a reactivation of CMV could be confirmed with PCR-analysis of peripheral blood and after medication with zovirax^® ^(acyclovir), cymevene^® ^(gancyclovir) was used for therapy for 9 days (was heißt das genau?). Subsequently, the CMV and EBV-PCR was negative again.

Atypical lymphocytes were found in the peripheral blood. FACS- analysis showed no evidence of recurrent pre-B-ALL suggesting virus-induced alterations. A high percentage of activated T-cells and a marked shift of T-cell subpopulations (CD4/CD8: 0,03) were conspicuous. Histopathological examination of bone marrow trephine biopsy showed a moderately hypocellular bone marrow with borderline maturation abnormalities of erythro- and megakaryopoesis, reactive eosinophilia and increased siderin in histiocytic cells. No lymphocytic or lymphoblastic infiltrates were detectable. Overall, the histological picture was considered to be consistent with drug-induced bone marrow toxicity.

CT-scan showed splenic enlargement and cervical, axillary, inguinal and abdominal lymphadenopathy. The brain was normal with no evidence of tumor infiltration.

**Figure 1:**
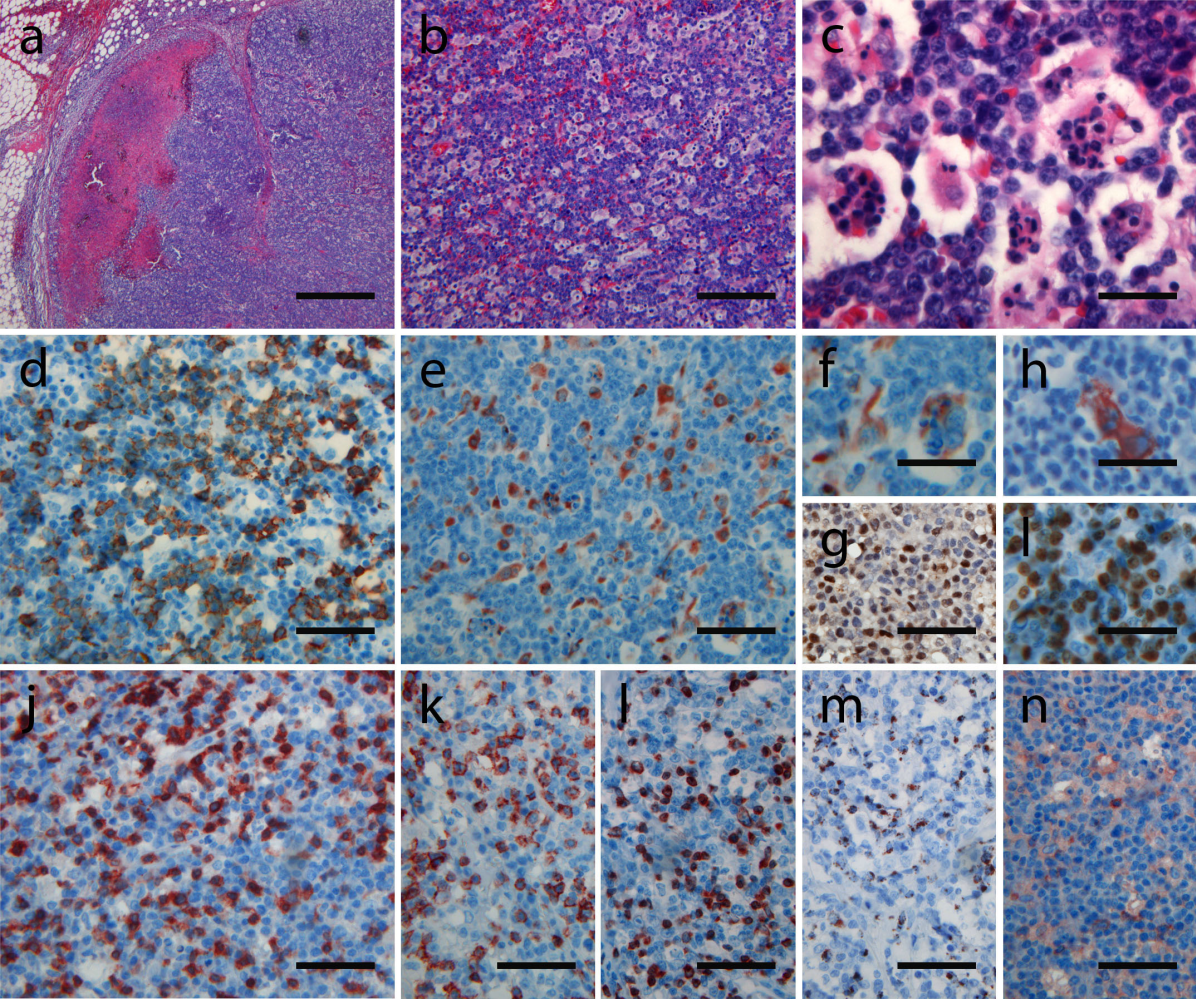
**On conventional HE staining a lymph node with vanished lymph node structure and focal necrosis and perinodal lymphatic infiltrates were seen (a)**. At higher magnification (b, c), a blastic infiltrate with strong apoptotic activity and many macrophages engulfing apoptotic nuclear bodes were visible. Immunolabeling confirmed the B-cellular lineage of most blastic cells (d: CD20) with macrophages cells lying in between (e+f: CD68). Part of the blastic cells was positive for EBNA2 (g) and EBV late membrane antigen (h: LMP). Overall, there was a high proliferative activity (i: Mib-1 positivity in about 80-90% of B-blasts). Additionally, a lot of CD3 (j)/CD8 (k) positive T-cells were interspersed, with hardly any CD4-positive T-helper cells identifiable (l). The T-cells were positive for perforin (m) and granzyme B (n). (a-c: hematoxylin eosin stainings; d-n: immunostainings with the antibodies indicated above) (magnification bars: a: 500 μm; b: 100 μm; c: 22 μm; d, e, g, i-n: 50 μm; f, h, l: 40 μm).

Cervical lymph node biopsy was performed 1 week after transferral to the hospital and 1 week subsequent to the reduction of immunosuppressive therapy. This revealed complete effacement of lymph node architecture (fig. 1a-c) due to infiltration by CD20-positive lymphoid blasts (Fig. 1d) with high proliferative activity (Mib-1 80-90%: fig. 1l). The vast majority of infiltrating cells were EBV-positive as demonstrated by EBER-specific in situ hybridisation (not shown). Scattered blast cells were positive for EBNA2 (fig. 1g) as well as LMP1 (fig. 1h), indicating a type III of EBV latency. PCR analysis showed a monoclonal rearrangement of the immunoglobulin heavy chain gene locus. Thus, the diagnosis of a monoclonal, monomorphic EBV-associated B-cell PTLD was made. Of note, focal areas of necrosis (fig. 1a: arrow head) and extensive apoptotic activity with many macrophages engulfing apoptotic nuclear bodies (fig. 1b, c, e, f) were found. The B-cell proliferation was accompanied by an extensive CD3-positive T-cell infiltrate (fig. 1j) consisting predominantly of CD8/perforin/granzymeB-positive cytotoxic T-cells (fig. 1k-m) with only very few CD4 positive cells present (fig. 1n). Chimerism testing showed that T-and B-cells, found in the lymph node, were 100% of donor origin.

CT-scans taken 1 week after biopsy showed regression of lymphadenopathy. Virological testing was finally negative for EBV and CMV. The patient achieved complete remission and was discharged 1 month after initial development of lymphadenopathy.

Two months later the patient was referred again to the hospital with diarrhoea and emesis, fever and shivering due to severe GvHD grade II. Despite intensive therapy the general condition of the patient worsened. He developed septicaemia and he became somnolent due to encephalopathy. He developed severe pulmonary oedema and died 12 months after initial diagnosis of ALL and 3 months after initial diagnosis of PTLD due to multi-organ failure.

At post-mortem examination, no residues of ALL or PTLD were found. The bone marrow was significantly hypocellular with a dramatic reduction of all three hematopoietic cell lines. Evidence of GvHD was found in stomach, small intestine and colon, and there were disseminated hyaline micro-thrombi in lungs and myocardium. Petechial bleeding was seen in small intestine, ileum and colon and there was extensive hemorrhage in the spleen. In addition, there was biventricular cardiac hypertrophy and evidence of congestive heart failure.

## Discussion

EBV-associated PTLD is an important complication of stem cell transplantation. In healthy individuals, primary EBV infection induces a virus-driven B-cell proliferation which may manifest clinically as infectious mononucleosis and which eventually is controlled by the development of a virus-specific T-cell immunity. This is directed against virus-encoded lytic and latent proteins and allows the establishment of life-long virus persistence in memory B-cells.

While virus persistence remains asymptomatic in the vast majority of individuals, occasionally EBV-associated tumours may develop, mostly of lymphoid origin. It is generally assumed that some degree of failure of the immune system to control EBV infection is involved in the pathogenesis of these neoplasms [5]. This failure may be in the microenvironment of the tumour cells. E.g., it is assumed that modulation of local immune reactions by cytokines produced in the tumour cells contributes to the development of EBV-associated Hodgkin lymphoma. On the other hand, failure of EBV-specific immunity may be systemic, as in transplant recipients subjected to iatrogenic immunosuppression [5]. The notion that failure of T-cell control of EBV infection is a crucial factor in the pathogenesis of PTLD is supported by several observations. PTLD cells frequently express EBV-encoded latent proteins, including EBNA2, which are recognised by EBV-specific T-cells in immunocompetent individuals [6]. Moreover it has long been known that PTLDs may regress upon reduction or withdrawal of immunosuppressive therapy [7]. Also, EBV-specific cytotoxic T-cells may induce regression of the outgrowth of EBV-transformed B-cells in vitro and this effect is abolished by cyclosporine A [8]. Finally, adoptive transfer of EBV-specific T-cells has proved successful in the treatment and prevention of PTLD [9].

Here we present, to our knowledge, the first description of the histopathological features of PTLD regression following reduction of immunosuppressive therapy. These were characterised by two main features. We demonstrate an intense infiltration of the affected lymph node by cytotoxic T-cells expressing CD8, perforin, and granzymeB. In addition, we observed extensive apoptosis of blast cells. The resulting apoptotic nuclear bodies were phagocytosed by macrophages. The latter feature is reminiscent of, e. g., Burkitt lymphoma and in germinal centre reactions, where a a high cellular turnover is accompanied by intense apoptotic activity. Although we cannot prove this directly, in the context of previous studies cited above, our results strongly suggest that apoptotic regression of the neoplastic EBV-positive B-cells was triggered by the re-emergence of EBV-specific T-cells in this case. This notion is well in line with current understanding of EBV-specific T-cell immunity [10]. In contrast, e. g. in Burkitt lymphoma a high spontaneous apoptotic rate is observed in the absence of cytotoxic T-cells. This might be related to the absence of antiapopotic factors such as bcl-2, which typically is not expressed in Burkitt lymphoma. Similarly, in physiological germinal centre reactions, in the absence of bcl-2 expression apoptosis is triggered by a lack of survival signals rather than cytotoxic activity of T-cells.

This role of cytotoxic T-cells in the control of EBV infection explains, why T-cell depletion via antithymocyte globulin or anti-CD3 monoclonal antibodies represents a major risk factor for the development of EBV-associated PTLD [4,11-13]. As the use of HLA-mismatched donor transplants usually requires more intensive immunosuppression, this additionally increases the risk for developing PTLD. Also, children and teenagers are at high risk of developing PTLD [14], most likely because an adequate primary immune response to EBV infection cannot be mounted under immunosuppressive therapy and because patients of this age group are frequently EBV seronegative before transplantation [15].

Although regression of PTLD following reduction or withdrawal of immunosuppressive therapy has been reported previously, the prognosis generally remains poor and treatment now includes rituximab and chemotherapy [16,17]. In our case, reduction of immunosuppression without further treatment led to a reduction of lymphadenopathy with full clinical remission accompanied by the development of GvHD. Patients with PTLD following stem cell transplantation usually die from progressive EBV-associated lymphoproliferation, sepsis, severe GvHD or relapse of the underlying haematological malignancy [18]. In our case, combined sepsis and GvHD were the leading causes of death and post mortem examination confirmed complete remission of PTLD.

In summary, we present a case of a case of PTLD after stem cell transplantation with complete remission following reduction of immunosuppressive therapy. We were able to show for the first time to our knowledge the histomorphological features occurring in PTLD lymph nodes in this scenario which are characterised by apoptotic cell death of EBV-infected B-blasts triggered by cytotoxic-T-cells.

## Competing interests

The authors declare that they have no competing interests.

## Authors' contributions

AK collected the data and was the main author of the manuscript. AM helped with evaluting the data. WP and DN were the treating oncologist. NS contributed to writing up the manuscript. GN was the consiliary pathologist. TA was the senior supervisor of the work performed. All authors read and approved the final manuscript.
